# Dietary and Health Correlates of Sweetened Beverage Intake: Sources of Variability in the National Health and Nutrition Examination Survey (NHANES)

**DOI:** 10.3390/nu13082703

**Published:** 2021-08-05

**Authors:** Susan E. Swithers, G. R. Bonanno, Janet Figueroa, Jean A. Welsh, Allison C. Sylvetsky

**Affiliations:** 1 Department of Psychological Sciences, Purdue University, West Lafayette, IN 47907, USA; gbonanno@purdue.edu; 2 Department of Pediatrics, School of Medicine, Emory University, Atlanta, GA 30322, USA; janet.figueroa@emory.edu (J.F.); jwelsh1@emory.edu (J.A.W.); 3 Child Wellness Department, Children’s Healthcare of Atlanta, Atlanta, GA 30322, USA; 4 Department of Exercise and Nutrition Sciences, Milken Institute School of Public Health, The George Washington University, Washington, DC 20052, USA; asylvets@gwu.edu; 5 Sumner M. Redstone Global Center for Prevention and Wellness, Milken Institute School of Public Health, The George Washington University, Washington, DC 20052, USA

**Keywords:** low-calorie sweeteners, diet beverages, sugar, obesity

## Abstract

Recent studies using data from the National Health and Nutrition Examination Survey (NHANES) have used inconsistent approaches to identify and categorize beverages, especially those containing low-calorie sweeteners (LCS), also referred to as low-calorie sweetened beverages (LCSBs). Herein, we investigate the approaches used to identify and categorize LCSBs in recent analyses of NHANES data. We reviewed published studies examining LCS consumption in relation to dietary and health outcomes and extracted the methods used to categorize LCS as reported by the authors of each study. We then examined the extent to which these approaches reliably identified LCSBs using the Internet Archive Wayback Machine to examine beverage ingredients lists across three NHANES cycles (2011–2016). None of the four general strategies used appeared to include all LCSBs while also excluding all beverages that did not contain LCS. In some cases, the type of sweetener in the beverage consumed could not be clearly determined; we found 9, 16, and 18 of such “mixed” beverage identifiers in the periods 2011–2012, 2013–2014, and 2015–2016, respectively. Then, to illustrate how heterogeneity in beverage categorization may impact the outcomes of published analyses, we compared results of a previously published analysis with outcomes when “mixed” beverages were grouped either all as LCSBs or all as sugary beverages. Our results suggest that caution is warranted in design and interpretation of studies using NHANES data to examine dietary and health correlates of sweetened beverage intake.

## 1. Introduction

There is now general scientific consensus that excess consumption of sugary beverages (SBs) contributes to a variety of negative health outcomes including overweight, obesity, type II diabetes, and cardiovascular disease [1]. However, the relationship between consumption of low-calorie sweetened beverages (LCSBs) and diet and health outcomes remains controversial. One common approach to assess potential impacts of LCSB consumption on dietary (e.g., energy intake, sugar intake) and health-related (e.g., body mass index, glycemic responses) outcomes has been to compare these outcomes in people who report consuming LCSBs with those who do not report LCSB consumption. This approach has been used by several studies based on data from the National Health and Nutrition Examination Survey (NHANES). NHANES is a survey of a nationally representative sample of approximately 5000 people in the United States each year and is widely used to investigate associations between dietary intake and a variety of diet- and health-related outcomes [2]. Since 1999, NHANES has been a continuous survey, with NHANES data collected in 2-year cycles, after which, results are released with a delay of approximately 2–4 years. The component of NHANES most relevant for studying dietary intake is the What We Eat in America (WWEIA) survey, which uses the USDA Automated Multiple-Pass Method to collect self-reported dietary intake data, using 24 h dietary recalls (one conducted in person and one conducted by phone) [3].

The process of using WWEIA/NHANES data to examine beverage intake patterns or outcomes related to beverage intake appears straightforward. For each food and beverage item in a participant’s dietary recall, the amount reported (e.g., 12 fluid oz can of diet ginger ale) is converted to a standardized 100 g portion, and the item is associated with a specific 8-digit foodcode (e.g., 92410560) in the USDA’s Food and Nutrient Database for Dietary Studies (FNDDS) [3]. To facilitate linking the correct FNDDS foodcode with items reported in the dietary recall, each foodcode contains a “Main food description” (e.g., “Soft drink, fruit flavored, caffeine containing, diet”). Some foodcodes also contain an “Additional food description”, which may include information about specific brands; brand names are also sometimes included in the “Main food description.” Each FNDDS foodcode is associated with specific energy and nutrient values derived from the USDA National Nutrient Database for Standard Reference (SR). Separate versions of the FNDDS, each associated with separate versions of the SR, are generated for each 2-year WWEIA/NHANES cycle. In the period 2011–2012, FNDDS contained 7618 foodcodes tied to SR 24 [4]; FNDDS 2013–2014 contained 8536 foodcodes linked to SR 26 [5]; and 8690 foodcodes associated with SR 28 were listed in FNDDS 2015–2016 [6].

Because FNDDS foodcodes serve as the basis for all nutrient composition data, a critical step in analyses of associations between LCSB intake and dietary and/or health outcomes is to determine how to categorize each foodcode into a specific beverage group. However, there is no information in WWEIA/NHANES/FNDDS that provides a clear, straightforward, or universally accepted way to reliably categorize which individual foodcodes represent which beverage types (i.e., sugar-sweetened beverages [SB] or low-calorie sweetened beverages [LCSB]), and different authors therefore have likely adopted different approaches to categorizing beverages in NHANES. At present, it is not clear how many different approaches have actually been used to identify and characterize beverages in WWEIA/NHANES, nor is it obvious how different approaches might impact the outcomes observed.

The primary goal of this study was to examine and describe approaches recently used to categorize beverages using WWEIA/NHANES data. We hypothesized that there would be broad heterogeneity in the approaches used to identify and categorize LCSBs, and that the number of beverages classified within beverage groups would vary substantially across studies, potentially leading to widespread misclassification of sweetened beverages. We also aimed to provide an example of how changing beverage foodcode classifications and correcting errors in WWEIA/NHANES can affect the outcomes of analyses, by re-analyzing previously published findings [7] from our group. We conducted this study in three separate phases. Because the results of each phase affected the methods of the subsequent phase, we describe the methods and results for each phase separately below.

## 2. Phase 1

### 2.1. Methods—Phase 1

In the first phase, we reviewed the existing literature to determine how different authors have identified beverages categorized as LCSBs. To identify approaches used to categorize beverages in prior analyses, we examined papers that used WWEIA/NHANES to examine dietary and health correlates associated with LCSB intake that were published between 2014 and 2019. The papers included met the following criteria: (1) relied on WWEIA/NHANES/FNDDS data; (2) included at least one beverage group identified as diet, low-calorie, and/or no-calorie; (3) described how diet, low-calorie and/or no-calorie beverages were identified; and (4) evaluated dietary intake across demographic groups and/or time. The papers included were not intended to be exhaustive, but instead to illustrate different approaches recently used for classification and analysis of LCSBs. Here, we refer to all groupings that included diet beverages or beverages containing LCS as LCSBs, although other terminology may have been used by the individual authors. For each paper, we determined what strategies and criteria were used to identify and group specific FNDDS foodcodes into beverage categories; whether a list of specific foodcodes used in the analysis was provided in the publication (either in the paper or Supplemental Material); whether specific foodcodes included in each beverage category were provided; and whether unsweetened beverages (such as coffee, tea and waters) were included in the LCSB group.

### 2.2. Results—Phase 1

Our examination of recent studies using WWEIA/NHANES data identified four general types of strategies for grouping LCSBs. Characteristics of analyses that employed each of these strategies are described in Table 1.

#### 2.2.1. Strategy 1: Organizational Structure of FNDDS

The organizational structure of the FNDDS foodcodes facilitates sorting beverages into groups and has been used to identify LCSBs in multiple studies. For example, the first 3 or 4 digits of the 8-digit foodcode are used to classify items in broad food groups and subgroups. In the period 2011–2016 [4,5,6], the foodcodes referring to non-alcoholic beverages begin with 92, and codes beginning with 924 refer to carbonated soft drinks.

#### 2.2.2. Strategy 2: WWEIA Categories

In addition to the FNDDS numbering scheme, each of the thousands of individual foodcodes is also grouped into one of approximately 150 mutually exclusive WWEIA categories [3,23]. There is no WWEIA category that explicitly reflects beverages that contain LCS, but a “Diet Beverages” group does encompass three WWEIA categories “Diet Soft Drinks (code 7102),” “Diet Sport and Energy Drinks (7104)” and “Other Diet Drinks (7106). These three WWEIA categories have been used as proxies to identify LCSBs.

#### 2.2.3. Strategy 3: Caloric Density

Another strategy is to identify LCSBs based on caloric density, for example using U.S. Food and Drug Administration (FDA) definitions to identify low-calorie (<40 kcal/serving) or no-calorie (<5 kcal/serving) beverages [24].

#### 2.2.4. Strategy 4: Text-Based Searches of FNDDS Food Code Descriptions and Other Combined Strategies

A final approach to identify LCSBs is to search foodcode descriptions specific terms frequently associated with LCSBs (e.g., “with low/no calorie sweetener”, “sugar free”, “dietetic/low sugar,” “no sugar added”, “light or lite”, “sugar-free”, “sugar substitute”, “low-calorie sweetener”, “no-calorie sweetener”, “reduced sugar”, “less sugar”, or “zero calorie”) or to search beverage ingredients lists for the presence of low-calorie sweeteners themselves. These text-based approaches are sometimes combined with energy or sugar content criteria to produce customized coding algorithms.

## 3. Phase 2

### 3.1. Methods—Phase 2

In the second phase, we evaluated whether the beverage categorization strategies elucidated in Phase 1 effectively captured all or most beverages which contained LCS, while also excluding those without LCS. We also assessed the advantages and disadvantages of each strategy, along with similarities and differences in how each strategy influenced the categorization of specific beverage foodcodes. For each foodcode, descriptive information available in the “Main Descriptions” and “Additional Description” in FNDDS was used to determine whether the foodcodes for every individual sweetened beverage in three NHANES cycles (2011–2012; 2013–2014; and 2015–2016) contained LCS. For some foodcodes, the descriptions explicitly listed LCS (e.g., “Light orange juice beverage, 40–50% juice, lower sugar and calories, with artificial sweetener”); these foodcodes were considered to contain LCS. If neither the main or description clearly indicated the presence of LCS, we examined ingredients lists available online for any brand(s) listed in either the main or additional descriptions to determine whether or not LCS were present, an approach similar to that described by others [10,17]. FNDDS foodcodes and their descriptions change across WWEIA/NHANES cycles, therefore FNDDS foodcode descriptions and ingredients lists for each beverage listed as being included within a given foodcode were examined and labeled separately for each of the three WWEIA/NHANES cycles.

In addition, because there is a delay of at least 2 years between dietary data collection in NHANES and the release of the data for analysis, currently available ingredient lists for beverages may not accurately reflect ingredients in the beverage when it was actually consumed. Therefore, we located historical online ingredient information for brand name products associated with each foodcode using the Wayback Machine from the non-profit Internet Archive [25,26]. For each branded product, we retrieved ingredient information archived on a date contemporaneous with each of the NHANES cycles (i.e., once in 2011 or 2012; once in 2013 or 2014; and once in 2015 or 2016). Whenever possible, manufacturer’s sites were used as the source of ingredient lists. In some cases, archived ingredient lists from manufacturer’s sites could not be located during the relevant period (primarily due to use of Adobe Flash™ (Adobe, San Jose, CA, USA) which led to archival of landing pages but not the supporting pages containing detailed ingredient information). In these cases, information was obtained from images of ingredient lists with copyright dates during the relevant timeframe or from online vendor pages (e.g., Amazon.com or Walmart.com) archived during the relevant time and which provided ingredient lists. If archival ingredient lists could not be located for a specific cycle, then ingredient lists from years prior to and after that cycle were examined. If the ingredients list did not contain LCS in years just before and after a given cycle, the beverage was considered not to contain LCS during that cycle.

### 3.2. Results—Phase 2

#### 3.2.1. Advantages and Disadvantages of Current Categorization Approaches

The number of foodcodes classified as LCSBs in papers that used one of the four general strategies described above spans at least the range of 14–148; for some analyses, the specific number of foodcodes cannot be determined with certainty (Table 1). None of the strategies for identifying LCSBs described above reliably identified all foodcodes for beverages that contained LCS while also consistently excluding foodcodes for beverages that did not contain LCS, and there was inconsistent overlap in foodcodes that would be identified across different strategies. Table 2 illustrates examples of some ways in which each type of strategy failed, using the list of FNDDS foodcodes that begin with 9255, a subcategory designated in FNDDS as “Fruit juice drinks and fruit flavored drinks, low calorie” [5,6]. For each foodcode, Table 2 includes the FNDDS main and additional food descriptions; the associated WWEIA category; the caloric density of each specific beverage according to both FNDDS and the manufacturer data at the time dietary recalls were obtained; and specific LCS(s) listed in the ingredients at the time that the dietary recall data were collected. Table 2 encompasses NHANES/WWEIA cycles 2013–2014 and 2015–2016, since neither the FNDDS information nor the manufacturer information changed across these cycles for this set of foodcodes.

##### Numbers of LCSB Foodcodes Identified Using Strategy 1

FNDDS organization allows for relative ease in determining which beverages are included in analysis. The analysis by Rusmevichientong et al. [19] described using this approach to classify diet soft drinks in NHANES cycles spanning 2005–2012. No specific details were provided for determining which of the 35 foodcodes beginning with 924 were considered diet soft drinks, and neither the number of foodcodes nor their identity were reported. However, 14 of the 35 foodcodes beginning with 924 were described as sugar-free, reduced sugar, or sweetened with low-calorie or no-calorie sweetener in FNDDS 2013–2016 [5,6], thus this analysis likely included 14 foodcodes. Bleich et al. [9] reported 14 specific foodcodes as diet beverages in their analysis spanning NHANES 2007–2010, suggesting that this strategy was also used to identify LCSBs in their analysis. The FNDDS organization approach includes very few foodcodes, thus, only a few of the hundreds of beverage foodcodes which do contain LCSs are ultimately categorized as LCSBs. In fact, none of the foodcodes listed in Table 2 would be identified as “diet” beverages in analyses relying on FNDDS organization.

##### Numbers of LCSB Foodcodes Identified Using Strategy 2

The approach of relying on WWEIA categories provides clarity about which foodcodes are included, and includes additional foodcodes beyond just those labeled soft drinks (e.g., those associated with diet sport and energy drinks or other diet drink categories). For example, using WWEIA diet beverage categories would identify five foodcodes from Table 2 as LCSBs, those associated with the category “Other Diet Drinks.” However, this approach excludes at least one foodcode that would be included based on FNDDS organization (92410250—Carbonated water, sweetened, with low-calorie sweetener- FNDDS 2013–2016 [5,6]). This foodcode is in the WWEIA category “Enhanced or fortified water,” a category that also includes a number of other beverage foodcodes that contain LCS. Thus, despite inclusion of additional foodcodes compared to FNDDS organization approaches, WWEIA categories approaches still identify only 30 beverage foodcodes as LCSBs in each cycle and therefore may miss LCS-containing beverages. 

##### Numbers of LCSB Foodcodes Identified Using Strategy 3

The caloric density approach identifies significantly more foodcodes than the approaches based on WWEIA/FNNDS organization and numbering strategies. However, there is no consensus on what specific caloric density cut-offs should be applied and it is sometimes unclear whether caloric density approaches include foodcodes for unsweetened beverages in their analyses. As a result, the range of beverage foodcodes identified using caloric density approaches is large and uncertain. For example, we [18] identified a total of 107 foodcodes from WWEIA/NHANES 2009–2010 as low- or no-calorie beverages based on FDA criteria; foodcodes included not only diet soft drinks, flavored waters, diet energy drinks and diet sport drinks, but also unsweetened and artificially sweetened teas and coffee. In contrast, Maillot et al. [16] defined the category “Other non-caloric and low-calorie beverages or LCB” to include beverages containing < 50 kcal/240 g. It is unclear whether unsweetened beverages other than water were included in their LCB group. Using yet another set of criteria, Leahy et al. [15] defined LCSBs as beverages with < 6.7 calories/8 oz (237 mL), and they listed 33 individual foodcodes from NHANES 2001–2012 in their Table 1.

Using this strategy also requires that the caloric density values listed in FNDDS be accurate and it implies that caloric density per se is a reliable proxy for the presence or absence of LCS in a beverage. As illustrated in Table 2, neither of these assumptions is actually true. Table 2 lists 2 foodcodes that do not contain any LCS and using any of the caloric density criteria described above, those foodcodes would have been excluded from LCSB groups. However, none of the caloric density criteria would have included all of the foodcodes in Table 2 for beverages that did contain LCS. For example, only 4 of the foodcodes with LCS meet Leahy et al.’s [15] criterion of < 6.7 kcal/8 oz (although they list only 3 of these foodcodes in their Table 1). Three additional foodcodes in Table 2 meet FDA criteria for low-calorie beverages; an additional 3 foodcodes meet the criterion of <50 kcal/240 g used by Maillot et al. [16]. Thus, depending on what the criterion was, between 4 and 10 of these 17 foodcodes in Table 2 as would be considered LCSBs based on the data in FNDDS, but the energy values listed in FNDDS frequently do not match those provided by manufacturers. This means that foodcodes in Table 2 could be either erroneously excluded or included as LCSBs in analyses that applied criteria based on caloric density. For example, between 2013 and 2016, foodcode 92550040 contained 46 kcal/8 fl oz according to FNDDS, but only 10 kcal/8 fl oz according to the manufacturer [27,28]. As a result, according to FDA criteria, it would be an LCSB based on manufacturer information but not based on FNDDS information. In contrast, foodcode 92552020 is listed in FNDDS with 5 kcal/8 fl oz, but actually had 60 kcal/8 fl oz based on the last available manufacturer data. As a result, all of the caloric density-based approaches would have listed this foodcode as a LCSB despite its actual calorie content being well above the criterion according to the manufacturer. Table 2 also provides clear evidence that product calorie content (regardless of whether provided by the manufacturer or based on FNDDS) does not accurately predict whether or not a given product contains LCS.

##### Numbers of LCSB Foodcodes Identified Using Strategy 4

Strategies that rely on text-based searches include significantly more beverage foodcodes that contain LCS compared to other approaches. In fact, because the category description itself includes the term “low-calorie”, all 17 foodcodes listed in Table 2 might be included in LCSB groups using this text-based approaches. However, it is often unclear how many foodcodes were included using such approaches since some methods lack sufficient details to determine what criteria were employed. Further, there has been little consensus on which specific terms are included, or how searches for specific terms have been combined with other criteria. This has resulted in wide variance in the number of foodcodes reported as LCSBs using text-based approaches. For example, Ford et al. [13] described low/no-calorie beverages as diet beverages (49 foodcodes reported) along with tap, bottled and flavored waters (5 foodcodes reported) in an analysis spanning 2003–2012. Two additional papers defined LCSBs as items whose descriptions included specific terms (e.g., ”with low/no calorie sweetener”, “sugar free”, or “dietetic/low sugar”) or “if review of the Nutrition Facts Panel ingredients list included any of the FDA-approved LCSs” [10,17]. Neither the specific foodcodes that met these descriptions, nor the total number of those foodcodes was reported. Drewnowski and Rehm [12] used a “custom coding algorithm” to identify items containing LCS “based on their description, energy density (kcal/100 g), and total and added sugars content (g) per average consumption report;” they did not provide specific information about the values used for their energy or sugar criteria or report the number of foodcodes or their identities. In two recent publications [7,21], we identified foodcodes as LCSBs if the FNNDS main foodcode description contained terms associated with low-calorie sweeteners, such as “diet”, “dietetic”, “low-calorie”, “no sugar added”, “light or lite”, “sugar-free”, “sugar substitute”, “low-calorie sweetener”, “no-calorie sweetener”, “reduced sugar”, “less sugar”, “zero calorie”, or “no sugar added.” For cycles spanning 2009–2012, this approach identified a total of 136 LCSB foodcodes [21], with 148 LCSB foodcodes identified for NHANES cycles 2011–2016 [7], although we also did not report the number or the identity of those foodcodes in those publications. In addition to the wide variance in the number of foodcodes identified as LCSBs, like caloric density-based approaches, text-based approaches can incorrectly identify foodcodes as LCSBs when they do not in fact contain any LCS. For example, in Table 2 foodcodes 92552030 and 92550380 did not list any LCS in their ingredients between 2013 and 2016. While at least one of these two foodcodes was incorrectly included in LCSB groups in at least some previous analyses (e.g., [7,13,21]), it is impossible to know how frequently this type of error occurred, given that some publications do not report specific foodcode lists.

#### 3.2.2. Additional Challenges of Using WWEIA/NHANES/FNDDS in Studies of LCSBs and Dietary and Health Outcomes

The process of determining how individual foodcodes were categorized led to the discovery of aspects of WWEIA/NHANES/FNDDS that further challenge the ability to analyze effects of LCSBs and SBs, regardless of the beverage classification strategy used.

##### Ambiguous Foodcodes

Many foodcodes associated with sweetened beverages can refer to more than one branded beverage. For example, Table 3 lists the different branded beverages associated with a single foodcode in WWEIA/NHANES cycles 2013–2016 (92530610 “Fruit juice drink, with high Vitamin C”). In FNDDS, this foodcode is associated with the WWEIA category (7204) labeled “Fruit drinks” and reported consumption of any of one of these beverages would have resulted in an assigned energy value of 114 kcal/8 oz. Thus, this foodcode is not likely to have been considered an LCSB in analyses using any of the four strategies described above. However, at least some of these products contained LCS according to their ingredients lists. Moreover, according to manufacturer data, the energy content of these beverages ranged from 30 to 140 kcal/8 oz, meaning that at least some of the products met FDA criteria for low-calorie beverages. Further, even within specific branded products with the same name, both the caloric density and presence or absence of LCS could vary based on how the product was packaged. For example, manufacturer information indicated that certain bottles and cans of Minute Maid Fruit Punch contained sucralose while other sizes of bottles and cans did not; the sucralose-containing packages also contained fewer calories. For other products in Table 3 (e.g., Hawaiian Punch), the type of sweetener(s) varied based on flavor, but the energy density did not appear to differ by flavor or based on which LCS were included. We identified 9, 16, and 18 sweetened beverage foodcodes in FNDDS 2011–2012, 2013–2014, and 2015–2016, respectively, in which the foodcode mixed multiple branded beverages where some contained LCS and others did not. For these mixed foodcodes, it is not possible to unambiguously determine whether the beverage consumed by an individual did or did not contain LCS (nor whether it would meet caloric density criteria as an LCSB).

##### Temporal Changes

Most analyses include multiple WWEIA/NHANES cycles, but they rarely specify which version(s) of FNDDS were used to categorize beverage foodcodes reported in each cycle. There is also little discussion of whether or not determinations were made separately for each cycle. This can lead to ambiguity and inaccuracy regardless of the categorization strategy employed. For example, the WWEIA “diet beverage categories” contained 28–30 separate foodcodes in each cycle between 2009 and 2016. However, the specific foodcodes contained within each cycle differed, and a total of 41 separate foodcodes would need to be included to capture all of those listed within those categories across those cycles. In addition, different versions of FNDDS can have different descriptions of individual foodcodes, including differences in beverage brands. As a result, a foodcode could meet the criteria for classification as a SB in one cycle, but changes in the description of the foodcode or beverage formulations could result in the same foodcode meeting the criteria for categorization as mixed or LCSBs in subsequent cycles.

##### Calculating Water Intake

Capturing intake of plain, unsweetened water has also not been straightforward in analyses of sweetened beverage intake relying on WWEIA/NHANES/FNDDS. For example, three foodcodes for plain water are listed in FNDDS 2011–2016; these refer to tap water (94000100); bottled water (94100100); and baby water (94300100). For FNDDS 2011–2014, the complete description of foodcode 94100100 was “Water, bottled, unsweetened; plain; flavored; spring water, nonsparkling or still; mineral water, nonsparkling or still”. However, in the period 2015–2016, foodcode 94220100 which had referred to “Propel Zero Water” was discontinued and Propel Zero Water was added to the description of 94100100 (the code for “Water, bottled, unsweetened; plain; flavored; spring water, nonsparkling or still; mineral water, nonsparkling or still”). This is perplexing because Propel Zero is a flavored, sweetened, water beverage containing both sucralose and AceK. While there is an unflavored version of Propel Water that contains added electrolytes without any LCS or sugar, it is not called Propel Zero, it has its own separate foodcode, 94210100, and it is appropriately included in the WWEIA category for Enhanced or Fortified waters rather plain water.

Further, the amount of water intake can be estimated in WWEIA/NHANES in several ways. One strategy is to sum the amounts reported for each of the three foodcodes assigned to plain water described above. Alternatively, dietary data files for WWEIA/NHANES include variables that are calculated from dietary records (e.g., DR1_320Z and DR2_320Z) and that are described as “Total plain water drank yesterday—including plain tap water, water from a drinking fountain, water from a water cooler, bottled water, and spring water”. Presumably the value of the sum of the intakes for each of the three separate “water” foodcodes should be identical to the value returned for the variables (e.g., DR1_320Z and DR2_320Z) that comprise those three foodcodes. However, for a small number of dietary reports (~3% in 2011–2016 cycles), these sums do not match based on our calculations.

An additional complication is that neither of these measures of water intake include carbonated water, which encompasses three additional foodcodes separate from the three plain water foodcodes. The first carbonated water foodcode, 92410210, had the description “carbonated water, unsweetened; all flavors; club soda; Perrier; seltzer water; sparkling mineral water” in the period 2011–2016. In most analyses, it is not clear whether this foodcode was included or how it was characterized if it was included. For example, some analyses [13,14,18] included it in a flavored water group, even though the remaining foodcodes in the flavored water groups are sweetened (most typically containing LCS). In analyses that relied on FNDDS organization, it may have been considered a soft drink since its foodcode begins with 924; because it had 0 calories per serving, this foodcode may have been included as a LCSB. On the other hand, it is not included in a WWEIA soft drink category, but instead in the WWEIA “Flavored or carbonated water” category. We did not include this foodcode in our 2019 analysis of children and adolescents [7] nor was it listed in Table 1 of the Leahy et al. analysis [15]. The remaining two carbonated water foodcodes represent sweetened carbonated water in FNDDS 2011–2016. Foodcode 92410250 is described as “carbonated water, sweetened, with low-calorie or no-calorie sweetener”, and is therefore clearly identifiable as LCSBs. This foodcode, which is also in the WWEIA category representing “Flavored or carbonated water,” is included with flavored water rather than with LCSBs in some analyses [13,14,18], while other analyses, including our 2019 paper [7] and the Leahy et al. report [15], included this code as LCSB. The final foodcode for carbonated water, 92410110, is described as “carbonated water, sweetened; tonic water; quinine water; fruit flavors; Clearly Canadian Original, all flavors; Penafiel, all flavors) and listed in the same WWEIA “Flavored or carbonated water” category. In our 2015 and 2019 papers [18,21], we included this code in our SB (soda) group, but it is not listed in any group in the Ford et al. analysis [13] and is included in the flavored water group by Grimes et al. [14]. Based on information from the Wayback Machine, beginning in at least as early as 2014, some flavors of Peñafiel waters (e.g., Apple Mineral Spring Water) contained sucralose and neotame along with high fructose corn syrup [29], and the caloric content of the beverages in this group of sweetened carbonated “waters” ranged from 16 to 90 kcal/8 oz according to the manufacturer. Thus, in 2014–2016, at a minimum, foodcode 92410110 contained both SBs and LCSBs, depending on the brand and flavor of beverage consumed, and therefore belongs with the group of foodcodes we have identified as mixed.

##### Converting between Volume and Weight across Beverages

Another potential source of error is related to how dietary information is collected and how data are reported in FNDDS. During dietary recalls, participants report foods and beverages in quantities that are familiar to them as consumers (e.g., 1 cup, a 12 oz can) rather than in grams. However, WWEIA/NHANES databases report the nutrient content of items, including beverages, in grams. Thus, beverage volumes reported in fluid ounces have to be converted to grams; for water, this means converting from 29.6 g to one fluid ounce. However, the number of grams per fluid ounce varies with the density of the beverage, and beverages in WWEIA/NHANES vary significantly in density. Each version of FNDDS provides the specific conversion factors that were used to convert intake reported in fluid ounces to grams for that cycle of WWEIA/NHANES, and these conversion factors ranged from 15.9 to 37 in the period 2011–2012 and from 24 to 32 in the period 2013–2016. This is of particular concern for analyses of LCSBs and SBs since beverages sweetened with LCS are less dense than those sweetened with sugars, and conversion factors provided in FNDDS reflect such differences in density (e.g., 30 g/fl oz vs. 31 g/fl oz). If the correct conversion factors are not applied for each foodcode, any analysis founded on comparisons of volume intake will introduce a systematic bias in calculations of intake of LCSBs compared to SBs. It is not clear what conversion factor(s) were actually employed in any of the published analyses evaluated, but we did not employ this approach in our 2019 child and adolescent paper [7].

## 4. Phase 3

### 4.1. Methods—Phase 3

To illustrate how addressing issues identified in Phase 2 might impact analyses of outcomes related to LCSB intake, we made a number of adjustments to our previously reported analysis of dietary patterns in children and adolescents [7] who reported consumption of LCSBs, SBs, both LCSBs + SBs, or water using WWEIA/NHANES 2011–2016. In that analysis, children and adolescents aged 2 to 17 years of age with one reliable in-person dietary recall who did not have a physician diagnosis of diabetes were categorized into one of 4 groups. LCSB consumers were those who reported consuming at least 4 oz LCSBs but fewer than 4 oz SBs; SB consumers were those who reported consuming at least 4 oz SBs but fewer than 4 oz LCSBs; LCSB + SB consumers who reported consuming at least 4 oz of each type of beverage; and water consumers were those who reported consuming at least 4 oz of water but fewer than 4 oz LCSBs and fewer than 4 oz SBs. Energy and macronutrient intakes were determined using multivariable linear regression models in SAS 9.4 following complex survey procedures to account for NHANES survey design. Covariates included age, sex, race/ethnicity, family income, physical activity, and body mass index (BMI) percentile in Model 1; total energy intake was added to these as a covariate in Model 2. Non-normal model residuals were transformed until residuals achieved normality; untransformed least squares means (LSM) and standard errors were reported, but transformed outcomes were used for pairwise comparisons, adjusted for multiple comparisons. *P* values of less than 0.05 were considered statistically significant.

For the present study, we used the same statistical approach [7], but adjusted our strategy for labeling beverages and addressed the other potential sources of error identified in Phase 2. Determination of beverage classification was done by individually and separately examining each foodcode for each WWEIA/NHANES cycle to determine whether ingredient lists contemporaneous with the cycle during which it was reported contained LCS. Thus, foodcodes could be classified differently during different WWEIA/NHANES cycles if beverage formulations or foodcode descriptions changed. Any foodcode was classified as LCSBs if all beverages included under that foodcode contained at least one LCS in the ingredient list during that cycle, or if the foodcode description explicitly indicated the presence of a LCS. Using this strategy, any beverage that listed both LCS and sugar in the ingredients list would be classified as a LCSB. If some, but not all, beverages listed within a foodcode included LCS during a particular cycle, the foodcode was considered to be mixed. We identified 87 foodcodes as LCSB, 249 SB, and 9 mixed foodcodes in the period 2011–2012; 137 LCSB, 295 SB and 16 mixed foodcodes in the period 2013–2014; and 134 LCSB, 291 SB and 18 mixed foodcodes in the period 2015–2016 (Supplementary Materials, Table S1). To account for mixed beverage foodcodes, we ran two separate analyses. In one analysis, all of the mixed foodcodes were considered to be LCSBs, while in the second analysis, all of the mixed foodcodes were considered to be SBs. All beverage volumes were calculated using the appropriate foodcode-specific conversion factor from WWEIA/NHANES to convert from grams to fluid ounces for each foodcode in each cycle separately. Plain water intake was determined from the dietary questionnaire (i.e., DR1_320Z); thus, unsweetened carbonated water was not included in calculation of water intake. We did not attempt to correct for the inclusion of Propel Zero, a sweetened water beverage, in the FNDDS foodcode for plain water, nor did we attempt to correct any discrepancies between manufacturer and FNDDS nutrient content for any foodcodes.

### 4.2. Results—Phase 3

Sweetened beverage (SB and LCSB) foodcodes reported to be consumed most frequently and in the greatest volume by children and adolescents in WWEIA/NHANES 2011–2016, when mixed codes were considered to be LCSBs, are listed in Table 4. Many of these most frequently consumed foodcodes are SBs, but also include one foodcode (92530610) that was mixed throughout the period 2011–2016 along with another (92541010) that was mixed only during NHANES 2015–2016.

**Figure 1:**
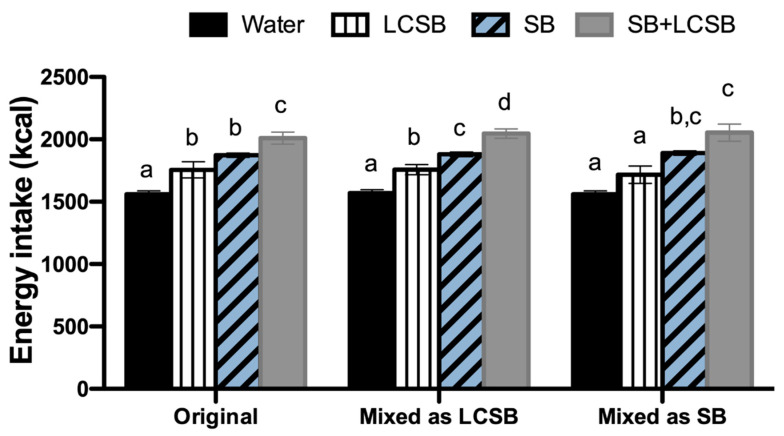
Energy intake after adjustment for age, sex, race/ethnicity, income, physical activity, and BMI percentile in original analysis [7] and re-analyses in which mixed foodcodes were considered LCSBs or SBs. Bars with different letters are significantly different from one another within their own analysis (i.e., groups were not compared across analyses).

**Figure 2:**
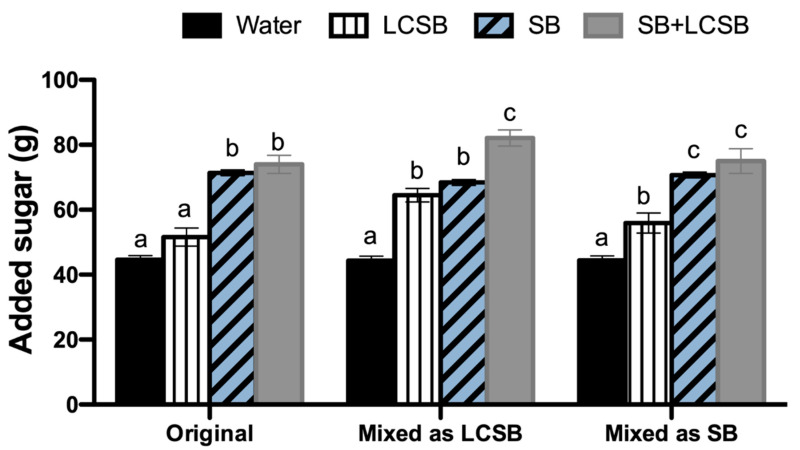
Added sugar intake after adjustment for age, sex, race/ethnicity, income, physical activity, BMI percentile, and energy intake in original analysis [7] and analyses in which mixed foodcodes were considered LCSBs or SBs. Bars with different letters are significantly different from one another within their own analysis (i.e., groups were not compared across analyses).

The largest consequence of addressing the issues identified and described in Phase 2, including changing whether mixed foodcodes were considered as LCSBs or SBs, was a change in the specific number of NHANES participants aged 2–17 who were categorized as consumers of LCSBs, SBs or both LCSBs + SBs (Table 5), compared to in our original analysis. In some cases, this impacted statistical comparisons between groups. For example, in our original analysis [7], no significant differences in total energy intake were observed between the LCSB and SB groups but energy intake was significantly greater in LCSB consumers compared to water consumers. In the present re-analysis, when mixed foodcodes were considered to be LCSBs, statistically significant differences in total energy intake were observed across all four groups (Figure 1 and Table 5). However, no differences in energy intake were observed between SB and LCSB + SB consumers, and both of these groups reported higher energy intake than LCSB and water consumers when mixed foodcodes were considered to be SBs. Further, in models adjusted for energy intake, carbohydrates and total sugar intake were significantly different across all four groups when mixed codes were considered LCSBs. In contrast, when mixed codes were considered SBs, outcomes were similar to our original results in which water and LCSB consumers had similar intakes which were significantly lower than in the SB and LCSB + SB groups. In contrast, added sugar intakes were significantly higher in the LCSB group compared with the water group irrespective of whether the mixed codes were considered LCSBs or SBs, but added sugar intakes were only lower in the LCSB group compared to the SB group when mixed codes were considered SBs (Figure 2). Consistent with our prior findings [7], mean estimates for total energy, total sugar, and added sugar intakes were highest in the LCSB + SB group. However, these results were not statistically significant for added sugar after adjusting for energy intake when mixed codes were considered SBs.

## 5. Discussion

Evidence on the relationship between the consumption of LCSBs and dietary or health-related outcomes based on data from NHANES has been inconsistent. This inconsistency may be explained, in part, by the issues we have described herein. First, reports that examine relationships between LCSB and dietary or health outcomes based on WWEIA/NHANES data have adopted heterogeneous approaches to categorize specific foodcodes into beverage groups, which has resulted in marked variability in the number of beverages that have been classified as LCSBs across analyses. Our results indicate that this number spans at least 14–148 FNDDS foodcodes, potentially representing hundreds of beverages. This range is concerning for a variety of reasons. For example, including a small number of foodcodes risks either excluding a large number of beverages that may contribute to dietary or health outcomes from analysis, or misclassifying LCSBs as SBs, and thereby muddying possible associations (or lack thereof). Analyses which include only 14 foodcodes as LCSBs adopt the simplest approaches which rely on either FNDDs foodcodes that begin with 9254 or rely on WWEIA soft drink categories. While this approach is convenient, our analysis shows that this approach would consider the effects of only 2 of the top 10 sweetened beverage foodcodes reported to be consumed by children and adolescents in 2011-2016. The remaining 8 foodcodes fall outside both the FNDDS foodcodes beginning with 9254 and the WWEIA soft drink categories. Analyses which have only considered carbonated soft drink foodcodes have thus failed to capture the impact of sweetened beverages that are consumed in large amounts, at least by children and adolescents.

One method to increase the likelihood that additional LCSBs will be included in analyses is to adopt criteria based on caloric density. Such an approach should be straightforward; nevertheless, we identified a number of issues that have complicated implementation and interpretation of this approach. First, the criteria applied have not been consistent across studies, and justification for the choice of criteria has not always been clear. One recommendation would be that analyses which use caloric density adhere to U.S. FDA definitions of low-calorie (<40 kcal/serving) or no-calorie (<5 kcal/serving) beverages, with the further specification that one serving is equal to 8 fluid ounces (or 237 mL). A second complication is that this strategy is more labor-intensive than it first appears because there is no simple method to separate unsweetened beverages from sweetened beverages using this approach. Nevertheless, consistent application of one definition of LCSB—a beverage that is sweetened and also low in calories—could be achieved with effort. However, this strategy is heavily reliant on the accuracy of the energy values reported in FNDDS, and our results indicate that there can be significant deviation between FNDDS values and those reported by manufacturers. Some of these concerns may be addressed with the ongoing development and release of databases containing manufacturer-derived information, such as the USDA Global Branded Food Products Database released with WWEIA/NHANES 2018–2019. However, given continued changes in beverage formulations, along with variance within the same beverage depending on flavor, packaging size, or serving option (i.e., packaged versus fountain), complete and useful information requires frequent and ongoing updates along with accurate documentation.

Using criteria based on caloric density does provide a strategy for identifying LCSBs that could be applied consistently, but not all sweetened beverages identified using FDA low-calorie criteria actually contain LCS. Thus, such an approach does not provide a method for identifying beverages which meet another definition of LCSB—that is, beverages which contain LCS, regardless of their caloric density. If an analysis aims to investigate relationships between beverages that contain LCS and dietary or health outcomes, then highly labor-intensive approaches which explicitly examine ingredient lists for the inclusion of LCS at the time of reported consumption must be employed. However, even such labor-intensive approaches cannot fully compensate for weaknesses in current FNDDS databases. Most problematic is the inclusion of multiple branded beverages within single foodcodes, some of which contain LCS and some of which do not (and which may or may not meet FDA low- or no-calorie definitions). Such mixed foodcodes require that multiple analyses be performed to permit understanding of the extent to which they could play a role. As demonstrated with our analysis of children and adolescents, group sizes and the magnitude of observed effects are indeed affected by whether the mixed foodcodes are considered to represent LCSBs or SBs. Disambiguating such foodcodes in future versions of WWEIA/NHANES would be an important improvement.

A critical question is what might explain the marked variability in beverage group definitions used in previous analyses. To some extent, these varied approaches likely reflect intentional design differences based on specific research questions. For example, some analyses are explicitly interested in all low- or no-calorie beverages, regardless of whether such beverages are sweetened or not. In other cases, the intent appears to be to examine specific beverages that are sweetened and low in calories, regardless of the type of sweetener. A third goal has been to examine all beverages that contain LCS. Conflating different research questions has contributed to confusion and lack of consensus. Such apparent lack of consensus is not unique to interpreting the effects of LCSBs on dietary and health outcomes from NHANES, but instead relates to broader concerns with a variety of social science approaches that have been criticized for lack of rigor, as evidenced by the divergent approaches adopted by different investigators when analyzing the same set of data in other disciplines [30]. Clarifying the specific question to be addressed and the measures used to address the questions should reduce variance and improve consensus [30].

In the context of WWEIA/NHANES/FNDDS and LCSBs, a lack of methodological detail precludes consensus since it is impossible to determine exactly how many foodcodes were included or how each foodcode was classified. Most analyses have also not specified the WWEIA/NHANES cycle(s) used for classification purposes, and none has explicitly indicated that classification of a specific foodcode could change over time. Finally, even when total numbers of foodcodes has been reported, the vast majority of publications do not report which specific foodcodes were included in which groups. As a result, determining how to compare outcomes across studies that purport to compare LCSBs with other types of beverages is nearly impossible. Clear and specific lists of foodcodes, along with how they were labeled and grouped in each WWEIA/NHANES cycle should be included to facilitate comparisons in analyses.

A second, and not mutually exclusive, possibility is that the lack of consensus is not an unintentional by-product of unclear research questions or measures, but instead may reflect an intentional strategy to support a certain outcome or minimize the likelihood that similar studies produce consistent results. An apparent lack of scientific consensus reduces the ability of scientists, the public, and policy makers to adopt actions that might improve health but which would adversely affect beverage sales and profits. As a result, groups with vested interests in promoting beverage intake could adopt confusing, misleading or inappropriate criteria and strategies either intended to support a specific outcome or to promote confusion. Such tactics have been described and adopted not only, for example, by the tobacco industry [31], but also by the beverage industry, consistent with evidence that beverage industry-funded studies are significantly more likely to report conclusions that serve the beverage industry’s interests [32,33,34,35]. While the contribution of this more insidious possibility to heterogeneity in approaches used to understand the impact of LCSBs remains unclear, this could also be improved by refining research questions and improving the clarity and transparency of the measures used to address those questions.

The critical importance of clarifying how sweetened beverages are defined for analyzing dietary exposures in relation to dietary and health outcomes has been highlighted in a recent report based on data from Switzerland. In that report, estimates of the intake of sweetened beverages varied from 240.6 g/day to 329.7 g/day depending how sweet beverages were defined [36]. Further, the current results demonstrate that the magnitude of the observed differences in estimates for both total and added sugar intakes when mixed foodcodes are classified as LCSBs compared to SBs is striking, even though the overall conclusions of our original analysis were not greatly affected. In light of the current limitations in accurately identifying beverages within FNDDS and the consistent lack of detail in previously published work on how specific foodcodes were classified, careful consideration in the interpretation of existing studies as well as in the design of future analyses of sweetened beverage intake based on NHANES data is warranted.

## Figures and Tables

**Table 1:** Characteristics of LCSB/Diet/Low-Calorie Beverage Groups from Selected Analyses.

Reference	NHANES Cycles Used	Foodcode Identity Reported	Number of Foodcodes	Unsweetened Beverages Included	Strategy
An, 2015 [8]	2003–2012	No	25 *	No	4
Bleich et al., 2014 [9]	2001–2010	No	14 *	No	1
DellaValle et al., 2018 [10]	2007–2012	No	Unknown	No	4
Demmer et al., 2018 [11]	2011–2014	No	31 **	No	2
Drewnowski and Rehm, 2015 [12]	1999–2008	No	Unknown	Unknown	4
Ford et al., 2016 [13]	2003–2012	Yes	54 *	Yes	4
Grimes et al., 2017 [14]	2005–2012	Yes	28 *	No	2
Leahy et al., 2017 [15]	2001–2012	Yes	33 *	No	3
Maillot et al., 2019 [16]	2011–2016	No	Unknown	Unknown	3
Malek et al., 2018 [17]	2007–2012	No	Unknown	No	4
Mesirow and Welsh, 2015 [18]	2001–2010	Yes	107 ***	Yes	3
Rusmevichientong et al., 2018 [19]	2005–2012	No	14 **	No	1
Shriver, 2018 [20]	2005–2012	No	Unknown	No	2
Sylvetsky, 2017 [21]	2009–2012	No	136 ***	No	4
Sylvetsky, 2019 [7]	2011–2016	No	148 ***	No	4
Watowicz, 2014 [22]	2005–2010	No	Unknown	Unknown	2

Foodcode identity reported—a value of “yes” indicates publications which provided a list of specific foodcodes included within each of their beverage groups. Number of foodcodes—values represent the number of foodcodes included in LCSB groups as reported by authors *; as estimated based on descriptions in manuscript **; or as known (but not previously reported) for publications from our group ***. “Unknown” indicates that numbers were not provided and could not be estimated based on descriptions. Unsweetened beverages included—a value of “yes” indicates publications in which LCSBs and unsweetened beverages were combined in a single group; “no” indicates that LCSB groups did not contain unsweetened beverages and “unknown” indicates that it could not be determined whether LCSB groups included unsweetened beverages. Strategy: 1—FNDDS Organization; 2—WWEIA categories; 3—Caloric Density; 4—Text-based or combined. See text for full descriptions of these strategies.

**Table 2:** Foodcodes in FNDDS category “Fruit juice drinks and fruit flavored drinks, low calorie” 2013–2016.

Foodcode	WWEIA Category	Main Food Description	Brands	LCS (as Listed by Mfr)	Kcal/8 oz	
					Mfr	FNDDS
92550030	7204—Fruit drinks	Fruit juice drink, with high vitamin C, light	Light Hawaiian Punch	Sucralose, AceK	10	46
			Minute Maid Light juice drinks	Aspartame, AceK	15	
			Tropicana Light lemonade	Sucralose * Aspartame, AceK *	* 10 * 5	
92550035	7204—Fruit drinks	Fruit juice drink, light	Minute Maid Light fruit punch **	Aspartame, AceK **	15	94
			Sunsweet prune juice light **	Sucralose **	100	
			Plumsmart^TM^ Light plum juice **	** Sucralose **	60	
92550040	7204—Fruit drinks	Fruit juice drink, diet	Diet Snapple juice drinks, all flavors	Aspartame	10	46
92550110	7204—Fruit drinks	Cranberry juice drink, with high vitamin C, light	Ocean Spray Light Cranberry Juice Cocktail	Sucralose	50	46
			Apple and Eve Light Cranberry juice drinks	Sucralose, AceK	10	
92550200	7204—Fruit drinks	Grape juice drink, light	Welch’s Light juice drinks	Sucralose, AceK	45	50
92550350	7204—Fruit drinks	Orange juice beverage, 40–50% juice, light	Tropicana Trop 50	RebA	50	50
			Minute Maid Light	Sucralose, AceK	50	
			Dole Light	***	***	
92550360	7204—Fruit drinks	Apple juice beverage, 40–50% juice, light	Tropicana Trop 50	RebA	50	55
			Mott’s Light	Sucralose	50	
92550370	7204—Fruit drinks	Lemonade, fruit juice drink, light	Tropicana Trop 50 Lemonade	RebA	50	52
92550380	7204—Fruit drinks	Pomegranate juice beverage, 40–50% juice, light	POM Lite, all flavors	None	75	130
92550400	7106—Other diet drinks	Vegetable and fruit juice drink, with high vitamin C, diet	Diet V8 Splash, all flavors, low calorie	Sucralose, AceK	10	10
92550405	7204—Fruit drinks	Vegetable and fruit juice drink, with high vitamin C, light	V8 V-Fusion Light, all flavors	Sucralose	50	10
92550610	7106—Other diet drinks	Fruit flavored drink, with high vitamin C, diet	Diet Ocean Spray cranberry, blueberry, or pomegranate blends	AceK, Sucralose	5	5
92550620	7106—Other diet drinks	Fruit flavored drink, diet	Crystal Light	Sucralose, AceK	5	10
			Minute Maid Light	Aspartame, AceK	15	
92552000	7106—Other diet drinks	Fruit flavored drink, with high vitamin C, powdered, reconstituted, diet	Sugar Free Tang (On-the-Go)	Aspartame, AceK	5	5
			Country Time Lite lemonade ^	Sucralose, AceK, Neotame ^	35 ^	
			Ocean Spray drink mix, low calorie	Aspartame, AceK	5	
92552010	7106—Other diet drinks	Fruit flavored drink, powdered, reconstituted, diet	Crystal Light ^^	Aspartame, AceK ^^ Rebiana ^^	5–15 ^^	5
			Sugar Free Kool-Aid	Aspartame, AceK	5	
			Wyler’s Light	Aspartame, AceK	5	
92552020	7204—Fruit drinks	Sunny D, reduced sugar	Reduced Sugar Sunny Delight fruit juice drink, all flavors ^	Sucralose, AceK, Neotame ^	60 ^	5
92552030	7204—Fruit drinks	Capri Sun, fruit juice drink	Capri Sun, NFS, 25% less sugar	None	94	97

Mfr—manufacturer, AceK—Acesulfame Potassium, RebA and Rebiana—Rebaudioside A. * Tropicana Light lemonade contained sucralose in packaged form but Aspartame and AceK from fountain. ** No brand identified in FNDDS, but Minute Maid light fruit punch, Sunsweet Plumsmart^TM^ light, and Sunsweet light prune juice met this definition (Light fruit juice drink; reduced sugar); *** Unable to locate product. ^ Product likely discontinued at this time—LCS from last known ingredients. ^^ Crystal light contained Aspartame and AceK while Crystal Light Pure and Crystal Light On-the-Go contained Rebiana.

**Table 3:** Branded beverages listed in the description of FNDDS Foodcode 92530610 (114 kcal/8 oz) “Fruit juice drink, with high Vitamin C” 2013–2016.

Brands Listed	LCS	Kcal/8 oz
Apple and Eve juice drinks	None	110
Florida’s Naturals juice cocktails	None	130
Hawaiian Punch	Sucralose, AceK *	60
	Sucralose *	60
Hi-C	None	110
Kool-Aid Jammers	Sucralose	30
Minute Maid	None **	110 **
	Sucralose **	90 **
Minute Maid Coolers	None	120
Ocean Spray juice drink or cocktail, flavors other than cranberry	None	110
Ssips	None	120
Tropicana fruit punch	None	130
Tropicana Lemonade, chilled carton	None	120
Tropicana Twister, all flavors except lemonade	None	140

* Hawaiian Punch Green Berry Rush flavor contained sucralose and AceK, while Fruit Juicy Red flavor contained only sucralose. ** Minute Maid Fruit had no LCS and 110 kcal/8 oz if sold in a 10 or 20 fl oz bottle, a 2 L bottle or a 12 fl oz can. If sold in a 128 fl oz bottle or 59 oz can, it contained sucralose and 90 kcal/8 oz.

**Table 4:** Sweetened beverage foodcodes reported to be consumed in the highest quantities by children and adolescents 2–17 years of age in NHANES 2011–2016 when “mixed” codes were considered LCSBs.

Rank	Foodcode	Description	WWEIA Category	SBs oz Reported	LCSBs oz Reported	Number of Reports
1	92410510	Soft drink, fruit flavored, caffeine free	7202 Soft drinks	10,455	-	994
2	92410310	Soft drink, cola	7202 Soft drinks	8355	-	763
3	64104010	Apple juice, 100%	7004 Apple juice	6668	-	864
4	92530610	Fruit juice drink, with high vitamin C	7204 Fruit drinks	-	6541	668
5	95320200	Gatorade G sports drink	7206 Sport and energy drinks	5420	-	345
6	92541010	Fruit flavored drink, powdered, reconstituted	7204 Fruit drinks	3430	1060	387
7	61210220	Orange juice, 100%, canned, bottled or in a carton	7002 Citrus juice	4284	-	546
8	61210250	Orange juice, 100%, with calcium added, canned, bottled or in a carton	7002 Citrus juice	3154	-	409
9	92552030	Capri Sun, fruit juice drink	7204 Fruit drinks	2796	-	353
10	64100110	Fruit juice blend, 100% juice	7006 Other fruit juice	2731	-	310

**Table 5:** Least square means and standard errors of energy and selected macronutrient intakes in US children and adolescents (2–17 y), NHANES 2011–2016, from original published analysis [7] compared with re-analyses in which all mixed foodcodes were categorized either as LCSBs or as SBs.

			Water ^1^	LCSBs ^2^	SBs ^3^	SBs + LCSBs ^4^
	Sample Size	Original [20]	*n* = 1077	*n* = 345	*n* = 4907	*n* = 697
		Mixed—LCSB	*n* = 1038	*n* = 589	*n* = 4288	*n* = 1093
		Mixed—SB	*n* = 1038	*n* = 273	*n* = 5160	*n* = 549
Energy (kcal)	Model 1	Original	1561 ± 27 ^a^	1756 ± 64 ^b^	1873 ± 18 ^b^	2010 ± 49 ^c^
		Mixed—LCSB	1569 ± 28 ^a^	1757 ± 41 ^b^	**1880 ± 18 ^c^**	**2046 ± 37 ^d^**
		Mixed—SB	1560 ± 27 ^a^	**1717 ± 70 ^a^**	**1891 ± 17 ^b,c^**	2054 ± 68 ^c^
Carbohydrates (g)	Model 1	Original analysis	192 ± 3.5 ^a^	222.3 ± 7.5 ^b^	252 ± 2.4 ^c^	271.9 ± 5.1 ^d^
		Mixed—LCSB	192.5 ± 3.7 ^a^	226.0 ± 5.1 ^b^	252.1 ± 2.5 ^c^	281.0 ± 4.5 ^d^
		Mixed—SB	191.3 ± 3.6 ^a^	**216.1 ± 8.8 ^a^**	**254.2 ± 2.3 ^b^**	**278.4 ± 7.7 ^c^**
	Model 2	Original analysis	228 ± 2.7 ^a^	235 ± 2.6 ^a^	251 ± 0.9 ^b^	255 ± 3.5 ^b^
		Mixed—LCSB	227.5 ± 2.8 ^a^	**239.0 ± 2.4 ^b^**	**250.4 ± 1.0 ^c^**	**259.8 ± 2.3 ^d^**
		Mixed—SB	227.4 ± 2.8 ^a^	233.8 ± 3.4 ^a^	251.2 ± 0.9 ^b^	256.3 ± 3.8 ^b^
Total Sugar (g)	Model 1	Original analysis	71.4 ± 2 ^a^	87.1 ± 3 ^b^	119.4 ± 1.5 ^c^	129.9 ± 3 ^d^
		Mixed—LCSB	72.3 ± 2.1 ^a^	96.0 ± 2.6 ^b^	118.7 ± 1.6 ^c^	137.5 ± 2.7 ^d^
		Mixed—SB	71.7 ± 2.1 ^a^	**86.8 ± 3.9 ^a^**	**120.1 ± 1.5 ^b^**	**133.8 ± 3.5 ^c^**
	Model 2	Original analysis	87.3 ± 1.6 ^a^	92.8 ± 3 ^a^	119 ± 0.9 ^b^	122.3 ± 3.5 ^b^
		Mixed—LCSB	87.8 ± 1.6 ^a^	**101.7 ± 2.4 ^b^**	**118 ± 1.0 ^c^**	**128.2 ± 2.5 ^d^**
		Mixed—SB	87.8 ± 1.6 ^a^	94.7 ± 3.7 ^a^	118.8 ± 0.9 ^b^	124.0 ± 3.7 ^b^
Added Sugar (g)	Model 1	Original analysis	33.2 ± 1.2 ^a^	47.6 ± 2.1 ^b^	71.7 ± 1.2 ^c^	79.4 ± 2.5 ^d^
		Mixed—LCSB	33.3 ± 1.4 ^a^	60.4 ± 2.1 ^b^	68.9 ± 132 ^c^	88.7 ± 2.7 ^d^
		Mixed—SB	32.9 ± 1.5 ^a^	50.3 ± 3.0 ^b^	71.7 ± 1.2 ^c^	82 ± 3.5 ^d^
	Model 2	Original analysis	44.6 ± 1.3 ^a^	51.6 ± 2.8 ^a^	71.4 ± 0.9 ^b^	74.0 ± 2.8 ^b^
		Mixed—LCSB	44.3 ± 1.4 ^a^	**64.5 ± 2.1 ^b^**	**68.4 ± 0.9 ^b^**	**82.1 ± 2.5 ^c^**
		Mixed—SB	44.4 ± 1.4 ^a^	55.9 ± 3.1 ^b^	**70.7 ± 0.9 ^c^**	**75.0 ± 3.7 ^c^**

Model 1: Adjusted for age, sex, race/ethnicity, income, physical activity, and BMI percentile. Model 2: Adjusted for age, sex, race/ethnicity, income, physical activity, BMI percentile, and energy intake. ^a,b,c,d^ Pairwise comparisons between the four consumer groups were Tukey adjusted. Different superscript letters indicate significant difference. If no superscript, then no significant differences between any of the groups. Post hoc outcomes in **bold** differ from original analysis. ^1^ Water: ≥ 4 oz water and < 4 oz of LCSBs, < 4 oz of SBs reported for day 1 recall, ^2^ LCSBs: ≥ 4 oz LCSBs and < 4 oz of SBs reported for day 1 recall, ^3^ SBs: ≥ 4 oz SBs and < 4 oz of LCSBs reported for day 1 recall, ^4^ SBs + LCSBs: ≥ 4 oz of both LCSBs and SBs reported for day 1 recall.

## Data Availability

NHANES data are publicly available at https://wwwn.cdc.gov/nchs/nhanes/continuousnhanes/default.aspx. Table S1 provides identification of sweetened beverage foodcodes and values used to convert fluid ounces to grams for NHANES 2011-216.

## Supplementary Materials

The following are available online at https://www.mdpi.com/article/10.3390/nu13082703/s1, Table S1: Identification of sweetened beverage foodcodes NHANES 2011–2016.

## References

[1] Hu F.B. (2013). Resolved: There Is Sufficient Scientific Evidence That Decreasing Sugar-Sweetened Beverage Consumption Will Reduce the Prevalence of Obesity and Obesity-Related Diseases. Obes. Rev..

[2] Centers for Disease Control and Prevention National Center for Health Statistics (Hyattsville, MD) NHANES Questionnaires, Datasets, and Related Documentation. https://wwwn.cdc.gov/nchs/nhanes/continuousnhanes/default.aspx.

[3] U.S. Department of Agriculture. A.R.S. WWEIA/NHANES Overview: USDA ARS. https://www.ars.usda.gov/northeast-area/beltsville-md-bhnrc/beltsville-human-nutrition-research-center/food-surveys-research-group/docs/wweianhanes-overview/.

[4] U.S. Department of Agriculture. A.R.S. USDA Food and Nutrient Database for Dietary Studies 2011–2012. https://www.ars.usda.gov/ARSUserFiles/80400530/pdf/fndds/fndds_2011_2012_doc.pdf.

[5] U.S. Department of Agriculture. A.R.S. USDA Food and Nutrient Database for Dietary Studies 2013–2014. https://www.ars.usda.gov/ARSUserFiles/80400530/pdf/fndds/fndds_2013_2014.pdf.

[6] U.S. Department of Agriculture. A.R.S. USDA Food and Nutrient Database for Dietary Studies 2015–2016. https://www.ars.usda.gov/ARSUserFiles/80400530/pdf/fndds/FNDDS_2015_2016_factsheet.pdf.

[7] Sylvetsky A.C., Figueroa J., Zimmerman T., Swithers S.E., Welsh J.A. (2019). Consumption of Low-Calorie Sweetened Beverages Is Associated with Higher Total Energy and Sugar Intake among Children, NHANES 2011-2016. Pediatr. Obes..

[8] An R. (2015). Beverage Consumption in Relation to Discretionary Food Intake and Diet Quality among US Adults, 2003 to 2012. J. Acad. Nutr. Diet.

[9] Bleich S.N., Wolfson J.A., Vine S., Wang Y.C. (2014). Diet-Beverage Consumption and Caloric Intake among US Adults, Overall and by Body Weight. Am. J. Public Health.

[10] DellaValle D.M., Malek A.M., Hunt K.J., St. Peter J.V., Greenberg D., Marriott B.P. (2018). Low-Calorie Sweeteners in Foods, Beverages, and Food and Beverage Additions: NHANES 2007–2012. Curr. Dev. Nutr..

[11] Demmer E., Cifelli C.J., Houchins J.A., Fulgoni V.L. (2018). Ethnic Disparities of Beverage Consumption in Infants and Children 0–5 Years of Age; National Health and Nutrition Examination Survey 2011 to 2014. Nutr. J..

[12] Drewnowski A., Rehm C.D. (2015). Socio-Demographic Correlates and Trends in Low-Calorie Sweetener Use among Adults in the United States from 1999 to 2008. Eur. J. Clin. Nutr..

[13] Ford C.N., Ng S.W., Popkin B.M. (2016). Ten-Year Beverage Intake Trends among US Preschool Children: Rapid Declines between 2003 and 2010 but Stagnancy in Recent Years. Pediatr. Obes..

[14] Grimes C.A., Szymlek-Gay E.A., Nicklas T.A. (2017). Beverage Consumption among U.S. Children Aged 0-24 Months: National Health and Nutrition Examination Survey (NHANES). Nutrients.

[15] Leahy M., Ratliff J.C., Riedt C.S., Fulgoni V.L. (2017). Consumption of Low-Calorie Sweetened Beverages Compared to Water Is Associated with Reduced Intake of Carbohydrates and Sugar, with No Adverse Relationships to Glycemic Responses: Results from the 2001-2012 National Health and Nutrition Examination Surveys. Nutrients.

[16] Maillot M., Vieux F., Rehm C.D., Rose C.M., Drewnowski A. (2019). Consumption Patterns of Milk and 100% Juice in Relation to Diet Quality and Body Weight Among United States Children: Analyses of NHANES 2011-16 Data. Front. Nutr..

[17] Malek A.M., Hunt K.J., DellaValle D.M., Greenberg D., St. Peter J.V., Marriott B.P. (2018). Reported Consumption of Low-Calorie Sweetener in Foods, Beverages, and Food and Beverage Additions by US Adults: NHANES 2007–2012. Curr. Dev. Nutr..

[18] Mesirow M.S.C., Welsh J.A. (2015). Changing Beverage Consumption Patterns Have Resulted in Fewer Liquid Calories in the Diets of US Children: National Health and Nutrition Examination Survey 2001-2010. J. Acad. Nutr. Diet..

[19] Rusmevichientong P., Mitra S., McEligot A.J., Navajas E. (2018). The Association between Types of Soda Consumption and Overall Diet Quality: Evidence from National Health and Nutrition Examination Survey (NHANES). Calif. J. Health Promot..

[20] Shriver L.H., Marriage B.J., Bloch T.D., Spees C.K., Ramsay S.A., Watowicz R.P., Taylor C.A. (2018). Contribution of Snacks to Dietary Intakes of Young Children in the United States. Matern. Child Nutr..

[21] Sylvetsky A.C., Jin Y., Clark E.J., Welsh J.A., Rother K.I., Talegawkar S.A. (2017). Consumption of Low-Calorie Sweeteners among Children and Adults in the United States. J. Acad. Nutr. Diet..

[22] Watowicz R.P., Taylor C.A. (2014). A Comparison of Beverage Intakes in US Children Based on WIC Participation and Eligibility. J. Nutr. Educ. Behav..

[23] U.S. Department of Agriculture, A.R.S. WWEIA Documentation and Data Sets: USDA ARS. https://www.ars.usda.gov/northeast-area/beltsville-md-bhnrc/beltsville-human-nutrition-research-center/food-surveys-research-group/docs/wweia-documentation-and-data-sets/.

[24] CFR—Code of Federal Regulations Title 21. https://web.archive.org/web/20100116095434/https://www.accessdata.fda.gov/scripts/cdrh/cfdocs/cfcfr/CFRSearch.cfm?fr=101.12.

[25] Wayback Machine. https://web.archive.org/.

[26] Arora S.K., Li Y., Youtie J., Shapira P. (2016). Using the Wayback Machine to Mine Websites in the Social Sciences: A Methodological Resource. J. Assoc. Inf. Sci. Technol..

[27] Diet Snapple Juice. https://web.archive.org/web/20140608083430/http://www.dpsgproductfacts.com:80/product/SNAPPLE_DIET_CRANBERRY_RASPBERRY_JUICE_DRINK_16.

[28] Dr Pepper Snapple Group Product Facts. https://web.archive.org/web/20160415035214/http://www.dpsgproductfacts.com/en/product/SNAPPLE_DIET_CRANBERRY_RASPBERRY_JUICE_DRINK_16.

[29] Dr Pepper Snapple Group Product Facts. https://web.archive.org/web/20141025205315/http://www.dpsgproductfacts.com:80/product/PENAFIEL_APPLE_MINERAL_SPRING_WATER_12.

[30] Auspurg K., Brüderl J. (2021). Has the Credibility of the Social Sciences Been Credibly Destroyed? Reanalyzing the “Many Analysts, One Data Set” Project. Socius.

[31] Brandt A.M. (2012). Inventing Conflicts of Interest: A History of Tobacco Industry Tactics. Am. J. Public Health.

[32] Schillinger D., Tran J., Mangurian C., Kearns C. (2016). Do Sugar-Sweetened Beverages Cause Obesity and Diabetes? Industry and the Manufacture of Scientific Controversy. Ann. Intern. Med..

[33] Nestle M. (2016). Corporate Funding of Food and Nutrition Research: Science or Marketing?. JAMA Intern. Med..

[34] Mandrioli D., Kearns C.E., Bero L.A. (2016). Relationship between Research Outcomes and Risk of Bias, Study Sponsorship, and Author Financial Conflicts of Interest in Reviews of the Effects of Artificially Sweetened Beverages on Weight Outcomes: A Systematic Review of Reviews. PLoS ONE.

[35] Lesser L.I., Ebbeling C.B., Goozner M., Wypij D., Ludwig D.S. (2007). Relationship between Funding Source and Conclusion among Nutrition-Related Scientific Articles. PLoS Med.

[36] Sousa A., Sych J., Rohrmann S., Faeh D. (2020). The Importance of Sweet Beverage Definitions When Targeting Health Policies-The Case of Switzerland. Nutrients.

